# Linear Program Relaxation of Sparse Nonnegative Recovery in Compressive Sensing Microarrays

**DOI:** 10.1155/2012/646045

**Published:** 2012-11-01

**Authors:** Linxia Qin, Naihua Xiu, Lingchen Kong, Yu Li

**Affiliations:** Department of Applied Mathematics, Beijing Jiaotong University, Beijing 100044, China

## Abstract

Compressive sensing microarrays (CSM) are DNA-based sensors that operate using group testing and compressive sensing principles. Mathematically, one can cast the CSM as sparse nonnegative recovery (SNR) which is to find the sparsest solutions subjected to an underdetermined system of linear equations and nonnegative restriction. In this paper, we discuss the *l*_1_ relaxation of the SNR. By defining nonnegative restricted isometry/orthogonality constants, we give a nonnegative restricted property condition which guarantees that the SNR and the *l*_1_ relaxation share the common unique solution. Besides, we show that any solution to the SNR must be one of the extreme points of the underlying feasible set.

## 1. Introduction

Nowadays, with the rapid development of molecular biology techniques, scientists use compressive sensing microarrays to collect the gene expression changes of patients suffer from specific diseases and test a lot of different drugs on cells genetically to look for medicine being able to change the abnormal gene expression [1, 2]. A DNA microarray is a collection of microscopic DNA spots attached to a solid surface. Each DNA spot contains a string of specific DNA sequences, known as probes. These can be a short section of a gene or other DNA element that are used to hybridize an organism's genetic sample under high-stringency conditions. Probe-target hybridization is usually detected and quantified by detection of chemiluminescence-labeled targets to infer the genetic makeup in the test sample.

Although the number of DNA sequences is extremely large, not all agents are expected to be present in a significant concentration at a given time and location. In traditional microarrays, this results in many inactive probes during sensing. On the other hand, we are often interested in only a small quantity of certain harmful biological agents. Therefore, it is important to not just detect the presence of agents in a sample but also estimate the concentrations with which they are present.

Assume that there are *m* spots and *n* labeled targets, and we have far fewer spots than target agents such that *m* ≪ *n*. Mathematically, one can represent the DNA concentration of each organism as an element in a vector *x* ∈ ℝ^*n*^ and the measurements as *b* ∈ ℝ^*m*^. For 1 ≤ *i* ≤ *m* and 1 ≤ *j* ≤ *n*, the probe at spot *i* hybridizes to target *j* with probability *a*_*ij*_. The target *j* occurs in the tested DNA sample with concentration *x*_*j*_, which is clearly nonnegative. Denoting by *A* : = (*a*_*ij*_)_*m*×*n*_, the process of DNA microarrays leads to the *sparse nonnegative recovery* (SNR) which is to find the sparsest solutions subjected to an underdetermined system of linear equations and nonnegative constraints, with the mathematical model as follows:

(*P*_0_) min||x||0, s.t. Ax=b, x≥0,

where the variable vector *x* ∈ ℝ^*n*^, ||*x*||_0_ denotes the number of the nonzero entries of *x*, *A* ∈ ℝ^*m*×*n*^ is the measurement matrix with full row rank, *m* ≪ *n*, and *b* ∈ ℝ^*m*^.

SNR can be regarded as a special case of the sparse recovery, which is related to program min{||*x*||_0_ | *Ax* = *b*}. This program has sparked the significant concern and rapid development in recent years [3–5] owing to its wide applications. However, with the nonnegativity prior information about the object to be recovered in various applications such as CSM, solutions on ((*P* _0_)) tend to be closer to the actual situations and lead to substantial improvements in the image reconstruction. Moreover, with the nonnegative constraints, the feasible set becomes a polyhedral set instead of an affine subspace. This will bring us essential hardness in projecting on the feasible set. Thus, ((*P* _0_)) is more likely difficult to solve. Therefore, SNR deserves specific study.

Problem ((*P* _0_)) has been shown to be NP-hard [6, 7] in general from the perspective of computational complexity. One popular approach is to reconstruct the vector via the *l*_1_ relaxation, which refers to

(*P*_1_) min||x||1, s.t. Ax=b, x≥0.

Since ((*P* _1_)) is a standard linear program, it is easy to solve. An important issue is how to guarantee the equivalence of ((*P* _0_)) and ((*P* _1_)) in the sense that they have the same unique *k*-sparse solution under some conditions. Here, we call a vector *x* *k*-sparse if the number of its nonzero entries is no more than *k*. There has been some increasing interest and activity in this area; see, for example, [8–14]. Donoho and Tanner [9] firstly proposed that ((*P* _0_)) and ((*P* _1_)) share the common *k*-sparse unique solution if the polytope *AT* is outwardly *k*-neighborly, where *T* is the standard simplex in ℝ^*n*^. Zhang [13] proved that ((*P* _0_)) and ((*P* _1_)) share the common *k*-sparse unique solution if the null space of *A* is strictly half *k*-balance. Juditsky et al. [11] developed several different necessary and sufficient conditions for the ((*P* _0_))-((*P* _1_)) equivalence in the case of general type sign restrictions, including the nonnegative constraints as its special case. When the feasible set of ((*P* _0_)) is a singleton, the unknown can be recovered by optimizing any objective function over this constraint set, and ((*P* _0_)) and ((*P* _1_)) definitely get the same unique solution. In this case, Bruckstein et al. [8] got the uniqueness of the feasible solution under a sufficient condition that *A* has a rowspan intersecting the positive orthant. Furthermore, Wang et al. [14] proved that the above sufficient condition is also necessary to the uniqueness of the feasible solution. Donoho and Tanner [10] proved that the underlying feasible set is a singleton if and only if the polytope *A*ℝ_+_^*n*^ and ℝ_+_^*n*^ have the same number of *k*-faces. Khajehnejad et al. [12] gave another equivalent condition of the uniqueness property by characterizing the support size of vectors in the null space of *A*.

For the *l*_1_ relaxation of sparse recovery, one of the most significant conditions is the restricted isometry property (RIP), named by Candès and Tao [15] with the groundbreaking work of Donoho et al. [16, 17]. However, to the best of our knowledge, the nonnegative case of RIP has not been investigated. This paper will deal with this issue. We begin with investigating the solution property of SNR and show that any solution to the SNR must be one extreme point of its feasible set in Section 2. We prove in Section 3 the nonemptiness and boundedness of the solution set of ((*P* _1_)) and show that any solution of ((*P* _1_)) could be stated as the convex combination of its optimal extreme points. In Section 4, by defining the nonnegative restricted isometry/orthogonality constants, we derive a sufficient condition for exact recovery of the sparsest nonnegative image/signal via the linear program relaxation.

Now we give some notations used in the text. We use sol(·) and *v*(·) to denote the solution set and optimal value of problem (·). The *e*_*i*_ ∈ ℝ^*n*^ would be the vector with only the *i*th entry 1 and the rest all 0. *e* ^*l*^ ∈ ℝ^*l*^ is the vector with each entry equal to 1; we also use *e* to demonstrate that *e* ^*n*^ ∈ ℝ^*n*^ for short. The *a*_*i*_ ∈ ℝ^*m*^ for *i* = 1,…, *n* denote the column vectors of the matrix *A* and *A*_*I*_ = (*a*_*i*_)_*i*∈*I*⊂{1,…,*n*}_. For any *x* ∈ ℝ^*n*^, *x*_*i*_ is the *i*th component and *I*(*x*) is the support set of *x*; that is, *I*(*x*) = {*i* | *x*_*i*_ ≠ 0, *i* = 1,…, *n*}. For any subset *T* ⊂ {1,…, *n*}, *T* ^*c*^ denotes the complement set of *T* out of {1,…, *n*}.

## 2. Solution Property

Throughout the paper we assume that

(1) $$\begin{array}{c} 𝒮:=\left\{x\in {\mathbb{R}}^{n}\;|\;Ax=b,x\geq 0\right\}\neq \emptyset . \end{array}$$

Apparently, *𝒮* is a polyhedral set in ℝ^*n*^. According to the representation theorem, any *x* ∈ *𝒮* could be represented as follows:

(2) $$\begin{array}{c} x=\sum_{i=1}^{t}\lambda_{i}{\overset{-}{x}}^{\left(i\right)}+\sum_{j=1}^{q}\mu_{j}d^{\left(j\right)}, \end{array}$$

where ∑_*i*=1_^*t*^ *λ*_*i*_ = 1, *λ*_*i*_ ≥ 0, *i* = 1,…, *t*, and $\left\{{\overset{-}{x}}^{\left(i\right)}\in {\mathbb{R}}^{n}|i=1,2,\ldots ,t\right\}$ are the extreme point set of *𝒮*; *μ*_*j*_ ≥ 0, *j* = 1,…, *q*, and {*d* ^(*j*)^ ∈ ℝ^*n*^ | *j* = 1,2,…, *q*} are the extreme direction set of *𝒮*. Apparently, $A{\overset{-}{x}}^{\left(i\right)}=b$, *i* = 1,…, *t*; *Ad* ^(*j*)^ = 0,  *d* ^(*j*)^ ≥ 0, *j* = 1,…, *q*.

Define subsets of ℝ^*n*^ as follows:

(3) S0=sub{0},S1={⋃i=1nsub{ei}}∖S0,S2={⋃i1,i2=1nsub{ei1,ei2}}∖⋃j=01Sj,   ⋮Sr={⋃i1,…,ir=1nsub{ei1,…,eir}}∖⋃j=0r−1Sj,   ⋮Sn={sub{e1,…,en}}∖⋃j=0n−1Sj,

where sub{*e*_*i*_1__,…, *e*_*i*_*k*__} denotes the subspace spanned by the vectors *e*_*i*_1__,…, *e*_*i*_*k*__, *k* = 1,…, *n*. Clearly, {*S*_1_, *S*_2_,…, *S*_*n*_} forms a partition of ℝ^*n*^; that is, ⋃_*j*=1_^*n*^ *S*_*j*_ = ℝ^*n*^ and *S*_*i*_⋂*S*_*j*_ = *∅*, *i* ≠ *j*. Moreover, ||*x*||_0_ = *r* for any *x* ∈ *S*_*r*_. Along with the nonemptiness of *𝒮*, it is easy to see that *𝒮* must intersect one of these sets, hence *v*(*P*_0_) = min{*r* ∈ ℝ | *𝒮*⋂*S*_*r*_ ≠ *∅*} and sol(*P*_0_) ≠ *∅*. Furthermore, we have the following result for the optimal value of ((*P* _0_)).

**Lemma 1**

Assume that *v*(*P*_0_) = *k*, one must have *k* ≤ *m*.

**Proof**

Suppose that the conclusion is not true; that is, there is x* ∈ sol(*P*_0_) with ||x*||_0_ = *k* > *m*. Without loss of generality, let *x*_i_* > 0, *i* = 1,…, *k*. We get

(4) $$\begin{array}{c} x_{1}^{*}a_{1}+\cdots +x_{k}^{*}a_{k}=b. \end{array}$$

Meanwhile, rank(*A*) = *m* < *k*. Thus, {*a*_*i*_ ∈ ℝ^*m*^ | *i* = 1,…, *k*} must be linearly dependent; that is, there exist *d*_1_,…, *d*_*k*_, not all zero, such that

(5) $$\begin{array}{c} d_{1}a_{1}+\cdots +d_{k}a_{k}=0. \end{array}$$

Assume that *d*_1_ > 0. By denoting *d* = (*d*_1_,…, *d*_*k*_, 0,…, 0)^*T*^ ∈ ℝ^*n*^, we get *Ad* = 0. Taking *δ* = min{*x*_i_*/*d*_*i*_ | *d*_*i*_ > 0, *i* = 1,…, *k*}, it holds that

(6) A(x∗−δd)=Ax∗−δAd=b,       x∗−δd≥0,      ||x∗−δd||0≤k−1.

This is a contradiction with x* ∈ sol(*P*_0_). We complete the proof.

To characterize property of the solution set sol(*P*_0_), we need the next lemma. In particular, this brand new result will play a key role in proposing the sufficient condition of the uniqueness of sol(*P*_0_) in Section 4.

**Lemma 2**

Any two distinct solutions of ((*P* _0_)) must have different support sets.

**Proof**

Assume for contradiction that x* and $\tilde{x}$ are two different solutions of ((*P* _0_)), $x^{*}\neq \tilde{x}$. If $I(x^{*})=I(\tilde{x})$, we have *x*_i_* > 0, ${\tilde{x}}_{i}>0$, for all *i* ∈ *I*(x*). Set $\lambda =\min\{\min_{i\in I(x^{*})}\{x_{i}^{*}/{\tilde{x}}_{i}\},\min_{i\in I(x^{*})}\{{\tilde{x}}_{i}/x_{i}^{*}\}\}$. Since $x^{*}\neq \tilde{x}$, it must hold *λ* < 1. When $\lambda =x_{i_{0}}^{*}/{\tilde{x}}_{i_{0}}$, take

(7) $$\begin{array}{c} \hat{x}=\frac{1}{1-\lambda }x^{*}-\frac{\lambda }{1-\lambda }\tilde{x}. \end{array}$$

It is easy to see that

(8) $$\begin{array}{c} {\hat{x}}_{i}=\frac{1}{1-\lambda }x_{i}^{*}-\frac{\lambda }{1-\lambda }{\tilde{x}}_{i}\in \{\begin{array}{cc} \left\{0\right\}, & i=i_{0},\\ {\mathbb{R}}_{+}, & i\in \;\;I\left(x^{*}\right)\setminus \left\{i_{0}\right\}. \end{array} \end{array}$$

Thus, we have $A\hat{x}=b$, $\hat{x}\geq 0$ and ${||\hat{x}||}_{0}\leq {||x^{*}||}_{0}-1$. This is a contradiction with the optimality of x*. When $\lambda ={\tilde{x}}_{j_{0}}/x_{j_{0}}^{*}$, just taking $\hat{x}=\left(1/\left(1-\lambda \right)\right)\tilde{x}-\left(\lambda /\left(1-\lambda \right)\right)x^{*}$ instead, we get the contradiction by a similar way. The proof is completed.

Now we are in a position to give the main theorem in this section.

**Theorem 3**

Any solution of ((*P* _0_)) must be one of the extreme points of *𝒮*.

**Proof**

Given any solution x* ∈ sol(*P*_0_) with representation

(9) $$\begin{array}{c} x^{*}=\sum_{i=1}^{t}\lambda_{i}^{*}{\overset{-}{x}}^{\left(i\right)}+\sum_{j=1}^{q}\mu_{j}^{*}d^{\left(j\right)}, \end{array}$$

where ∑_*i*=1_^*t*^ *λ*_i_* = 1, *λ*_i_* ≥ 0, ${\overset{-}{x}}^{\left(i\right)}\geq 0$, $A{\overset{-}{x}}^{\left(i\right)}=b$, for all *i* = 1,…, *t*; *μ*_j_* ≥ 0, *d* ^(*j*)^ ≥ 0, *Ad* ^(*j*)^ = 0, for all *j* = 1,…, *q*. We only need to prove that

(10) $$\begin{array}{c} \sum_{i=1}^{p}\lambda_{i}^{*}=1,\;\lambda_{i}^{*}\in \left\{0,1\right\},\;\;i=1,\ldots ,t,\\ \mu_{j}^{*}=0,\;j=1,\ldots ,q, \end{array}$$

in (9). To this end, we have the following three steps.

Firstly, we claim that in (9),

(11) I(x−(i))=I(x∗), i∈{i ∣ λi∗>0,  i=1,…,t},I(d(j))⊂I(x∗), j∈{j ∣ μj∗>0,  j=1,…,q}.

According to the fact that ${\overset{-}{x}}^{\left(i\right)}\geq 0$, *i* = 1,…, *t*, and *μ*_j_* ≥ 0, *d* ^(*j*)^ ≥ 0, *j* = 1,…, *q*, one has for any $l\in I({\overset{-}{x}}^{\left(i\right)})$, *i* ∈ {*i* | *λ*_i_* > 0, *i* = 1,2,…, *t*},

(12) $$\begin{array}{c} x_{l}^{*}=\sum_{i=1}^{t}\lambda_{i}^{*}{\overset{-}{x}}_{l}^{\left(i\right)}+\sum_{j=1}^{q}\mu_{j}^{*}d_{l}^{\left(j\right)}>0, \end{array}$$

which means that *l* ∈ *I*(x*). This implies $I({\overset{-}{x}}^{\left(i\right)})\subset I(x^{*})$. Similarly, we get *I*(*d* ^(*j*)^) ⊂ *I*(x*). On the contrary, from the optimality of x* and (9), we know that $I(x^{*})\subset I({\overset{-}{x}}^{\left(i\right)})$.

Secondly, we will show that *μ*_j_* = 0, *j* = 1,…, *q*. If this is not true, there is an index *j*_0_ such that *μ*_*j*_0__* > 0, then *μ*_*j*_0__*d ^(*j*_0_)^ has at least one positive component. Denote *d* = ∑_*j*=1_^*q*^ *μ*_j_*d ^(*j*)^, so *Ad* = 0 and {*d*_*l*_ > 0 | *l* = 1,…, *n*} ≠ *∅*. Noting that ${\overset{-}{x}}^{\left(i\right)}\geq 0$, (11) implies the fact that $I(\sum_{i=1}^{t}\lambda_{i}^{*}{\overset{-}{x}}^{\left(i\right)})=I(x^{*}),I(d)\subset I(x^{*})$. Take $\delta =\min\{{\left(\sum_{i=1}^{t}\lambda_{i}^{*}{\overset{-}{x}}^{\left(i\right)}\right)}_{l}/d_{l}|d_{l}>0,l=1,\ldots ,n\}$, and

(13) $$\begin{array}{c} \tilde{x}=\sum_{i=1}^{t}\lambda_{i}^{*}{\overset{-}{x}}^{\left(i\right)}-\delta d. \end{array}$$

Without loss of generality, set $\delta ={\left(\sum_{i=1}^{t}\lambda_{i}^{*}{\overset{-}{x}}^{\left(i\right)}\right)}_{l_{0}}/d_{l_{0}}$. It is easy to verify that

(14) $$\begin{array}{c} {\tilde{x}}_{j}={\left(\sum_{i=1}^{t}\lambda_{i}^{*}{\overset{-}{x}}^{\left(i\right)}\right)}_{j}-\delta d_{j}\in \{\begin{array}{cc} \left\{0\right\}, & j=l_{0}\\ {\mathbb{R}}_{+}, & i\in I\left(x^{*}\right)\setminus \left\{l_{0}\right\}, \end{array} \end{array}$$

hence

(15) Ax~=b, x~≥0,||x~||0≤||x∗||0−1,

which is a contradiction with the optimality of x*.

Thirdly, we will prove that ∑_*i*=1_^*t*^ *λ*_i_* = 1, *λ*_i_* ∈ {0,1}, *i* = 1,…, *t*. Suppose that there exist *λ*_1_* > 0, *λ*_2_* > 0, and *λ*_1_* + *λ*_2_* = 1 and two different extreme points of *𝒮*, say ${\overset{-}{x}}^{\left(1\right)},{\overset{-}{x}}^{\left(2\right)}$, such that

(16) $$\begin{array}{c} x^{*}=\lambda_{1}^{*}{\overset{-}{x}}^{\left(1\right)}+\lambda_{2}^{*}{\overset{-}{x}}^{\left(2\right)}. \end{array}$$

Based on (11), we have $I({\overset{-}{x}}^{\left(1\right)})=I({\overset{-}{x}}^{\left(2\right)})=I(x^{*})$, hence ${\overset{-}{x}}^{\left(1\right)}\in \text{ sol }\left(P_{0}\right)$, ${\overset{-}{x}}^{\left(2\right)}\in \text{ sol }\left(P_{0}\right)$. Nevertheless, by Lemma 2, this is impossible. Hence, we show that

(17) $$\begin{array}{c} x^{*}\in \left\{{\overset{-}{x}}^{\left(1\right)},{\overset{-}{x}}^{\left(2\right)},\ldots ,{\overset{-}{x}}^{\left(t\right)}\right\}. \end{array}$$

We complete the argument.

Theorem 3 tells us that each solution of ((*P* _0_)) lies in the extreme point set of *𝒮*. Here is a concrete example.

**Example 4**

Let $A=\left(\begin{array}{ccc} 1 & 1 & -3\\ -1 & 0 & 2 \end{array}\right),b=\left(\begin{array}{c} 3\\ -2 \end{array}\right)$. Obviously, the solution set and optimal value of ((*P* _0_)) are

(18) $$\begin{array}{c} \text{sol}\left(P_{0}\right)=\left\{\left(2,1,0\right)\right\},\;\;v\left(P_{0}\right)=2, \end{array}$$

respectively. In this case, (2,1, 0) is the only extreme point of *𝒮*. While the solution set and optimal value of (*P*) : = min{||*x*||_0_ | *Ax* = *b*} are

(19) $$\begin{array}{c} \text{sol}\left(P\right)=\left\{\left(0,0,-1\right)\right\},\;\;v\left(P\right)=1, \end{array}$$

respectively.

At the end of this section, we consider the *l*_*p*_ (0 < *p* < 1) relaxation of ((*P* _0_))

(*P*_*p*_) min ||x||pp=∑i=1nxip, s.t. Ax=b,   x≥0.

Clearly, ((*P* _*p*_)) is a concave relaxation of ((*P* _0_)). For the program ((*P* _*p*_)), Ge et al. [7] derived the useful result as the following.

**Lemma 5**

The set of all extreme points of *𝒮* is exactly the set of all local minimizers to ((*P* _*p*_)).

This lemma implies that any global solution of *l*_*p*_ relaxation must be one of its extreme points. From Theorem 3 and Lemma 5, we immediately draw a new proposition.

**Proposition 6**

For any *p* ∈ (0,1), there exists an extreme point of *𝒮* that is both an exact solution of ((*P* _0_)) and a local minimizer of ((*P* _*p*_)).

This is different from the result of Fung and Mangasarian in [18], where they showed that for sufficiently small $\overset{-}{p}\in (0,1)$, there exists an extreme point $\overset{-}{x}$ of the polyhedral set *𝒯*, obtained by lifting the set *𝒮*, such that $\overset{-}{x}$ is an exact solution of ((*P* _0_)) and a global solution of the $l_{\overset{-}{p}}$ relaxation.

## 3. Linear Program Relaxation

Consider the linear program relaxation ((*P* _1_)). Since the linear objective function 〈*e*, *x*〉 is bounded below over the feasible set, based on the Frank-Wolfe theorem, the minimum of ((*P* _1_)) is attainable. Among all the extreme points of *𝒮*, $\left\{{\overset{-}{x}}^{\left(i\right)}\in {\text{R}}^{n}|i=1,\ldots ,t\right\}$, we call ${\overset{-}{x}}^{\left(i\right)}$ an optimal extreme point if it also meets $\langle e,{\overset{-}{x}}^{\left(i\right)}\rangle =v\left(P_{1}\right)$.

**Proposition 7**

Any x* ∈ sol (*P*_1_) could be stated as the convex combination of optimal extreme points of ((*P* _1_)). Hence, sol (*P*_1_) is bounded.

**Proof**

Given any x* ∈ sol(*P*_0_) with representation (9). If there is *i*_0_ ∈ {1,…, *t*} such that ${\overset{-}{x}}^{\left(i_{0}\right)}$ is not an optimal extreme point of ((*P* _1_)) and *λ*_*i*_0__* > 0, we have $\langle e,{\overset{-}{x}}^{\left(i_{0}\right)}\rangle >v\left(P_{1}\right)$, hence

(20) v(P1)=〈e,x∗〉=∑i=1tλi∗〈e,x−(i)〉+∑j=1qμj∗〈e,d(j)〉≥∑i=1tλi∗〈e,x−(i)〉>∑i=1tλi∗v(P1)=v(P1),

which is a contradiction.

Similarly, if there is *j*_0_ ∈ {1,…, *q*} such that *μ*_*j*_0__* > 0, we have *μ*_*j*_0__*〈*e*, *d* ^(*j*_0_)^〉>0, hence

(21) v(P1)=〈e,x∗〉=∑i=1tλi∗〈e,x−(i)〉+∑j=1qμj∗〈e,d(j)〉>∑i=1tλi∗〈e,x−(i)〉≥∑i=1tλi∗v(P1)=v(P1),

which is a contradiction. This completes the proof.

From the above proposition, we know that linear program ((*P* _1_)) has at least one optimal extreme point. Thus, we could use simplex method or interior point method to solve ((*P* _1_)).

## 4. Nonnegative Restricted Property

In the framework of *l*_1_ relaxation, a significant problem is how to guarantee the exact recovery of sparse image/signal via the *l*_1_ relaxation. One of the most important qualifications is the restricted isometry property; see [15]. Recall that the *k-restricted isometry constants* (RIC) *δ*_*k*_ is the smallest scalar satisfying

(22) $$\begin{array}{c} \left(1-\delta_{k}\right){||x||}_{2}^{2}\leq {||Ax||}_{2}^{2}\leq \left(1+\delta_{k}\right){||x||}_{2}^{2},\;\forall {||x||}_{0}\leq k. \end{array}$$

Similarly, the *k*, *k*′*-restricted orthogonality constants* (ROC) *θ*_*k*,*k*′_ for *k* + *k*′ ≤ *n* is defined as the smallest scalar satisfying

(23) |〈Ax,Ay〉|≤θk,k′||x||2||y||2,    ∀||x||0≤k;  ∀||y||0≤k′,

where *x* and *y* have disjoint support sets. The RIC *δ*_*k*_ and ROC *θ*_*k*,*k*′_ measure how close each submatrix of *A* with certain cardinality is behaving like an orthonormal system. Under some restricted isometry property, one can get the sparse recovery via its *l*_1_ relaxation. Nevertheless, for the nonnegative case, the sparse recovery may maintain new characterizations. Above all, we define NRIC and NROC.

**Definition 8**

Let *A* ∈ ℝ^*m*×*n*^. We define the nonnegative *k*-restricted isometry constants (NRIC) *δ*_*k*_^+^ as the smallest number satisfying

(24) (1−δk+)||x||22≤||Ax||22≤(1+δk+)||x||22,            ∀x≥0,  ||x||0≤k.

Similarly, we define the nonnegative *k*, *k*′-restricted orthogonality constants (NROC) *θ*_*k*,*k*′_^+^ for *k* + *k*′ ≤ *n* as the smallest number satisfying

(25)      |〈Ax,Ay〉|≤θk,k′+||x||2||y||2,∀x≥0,||x||0≤k,∀y≥0,||y||0≤k′,

with *I*(*x*) and *I*(*y*) being disjoint sets.

Clearly,

(26) $$\begin{array}{c} \delta_{k}^{+}\leq \delta_{k},\;\theta_{k,k^{\prime }}^{+}\leq \theta_{k,k^{\prime }}. \end{array}$$

Moreover, the numbers *δ*_*k*_^+^ and *θ*_*k*,*k*′_^+^ are nondecreasing in *k*, *k*′.

By employing the projections of vectors in the null space of *A* to ℝ_+_^*n*^, we now provide a sufficient condition to determine a solution of ((*P* _0_)).

**Theorem 9**

Suppose that *k* ≥ 1 is such that *δ*_*k*_^+^ + *θ*_*k*,*k*−1_^+^ < 1 and x* ∈ *𝒮* with ||x*||_0_ = *k*. Then, x* is a solution of ((*P* _0_)).

**Proof**

We complete the proof by contradiction. If this is not true, there exists $\overset{-}{x}$ such that $A\overset{-}{x}=b$, $\overset{-}{x}\geq 0,{||\overset{-}{x}||}_{0}\leq k-1$. Set $h=x^{*}-\overset{-}{x}$. Clearly, *Ah* = 0. Take *h* = *h* ^+^ − *h* ^−^ with *h* ^+^ and *h* ^−^ being the projections of *h* and −*h* to ℝ_+_^*n*^, respectively. We have *h* ^+^ ≥ 0, *h* ^−^ ≥ 0, 〈*h* ^+^, *h* ^−^〉 = 0. In particular,

(27) $$\begin{array}{c} {\left(h^{+}\right)}_{i}=\{\begin{array}{cc} {\left(x_{i}^{*}-{\overset{-}{x}}_{i}\right)}_{+}, & i\in I\left(x^{*}\right),\\ 0, & \text{else}, \end{array}\\ {\left(h^{-}\right)}_{i}=\{\begin{array}{cc} {\left(x_{i}^{*}-{\overset{-}{x}}_{i}\right)}_{-}, & i\in I\left(\overset{-}{x}\right),\\ 0, & \text{else}. \end{array} \end{array}$$

Therefore,

(28) 1≤||h||0≤||x∗||0+||x−||0≤2k−1,1≤||h+||0≤k,0≤||h−||0≤k−1.

Thus, we get

(29) 0=||Ah||22=||Ah+−Ah−||22=||Ah+||22+||Ah−||22−2〈Ah+,Ah−〉≥(1−δk+)||h+||22+(1−δk−1+)||h−||22 −θk,k−1+(||h+||22+||h−||22)≥(1−δk+−θk,k−1+)||h+||22 +(1−δk−1+−θk,k−1+)||h−||22>0,

in which the first inequality is due to (24), (25), and the fact that 2*ab* ≤ *a* ^2^ + *b* ^2^, and the last inequality is because of the assumption of *δ*_*k*_^+^ + *θ*_*k*,*k*−1_^+^ < 1 and the monotonicity of *δ*_*k*_^+^ in *k*. This is a contradiction. Therefore, x* ∈ sol(*P*_0_).

With the special result that any two solutions of ((*P* _0_)) have different support sets, we next derive a sufficient condition on the uniqueness of solution to ((*P* _0_)).

**Theorem 10**

Suppose that *k* ≥ 1 is such that *δ*_*k*_^+^ + *θ*_*k*,*k*_^+^ < 1 and x* ∈ *𝒮* with ||x*||_0_ = *k*. Then, x* is the unique solution of ((*P* _0_)).

**Proof**

Since *δ*_*k*_^+^ + *θ*_*k*,*k*_^+^ < 1 implies *δ*_*k*_^+^ + *θ*_*k*,*k*−1_^+^ < 1, we know that x* ∈ sol(*P*_0_). Now we just need to verify that x* is the unique solution of ((*P* _0_)). Assume that this is not true; that is, there is another solution $\overset{-}{x}\neq x^{*}$. According to Lemma 2, it must hold $I(\overset{-}{x})\neq I(x^{*})$. Take $h=x^{*}-\overset{-}{x}$. By the argument similar to that in the proof of Theorem 9, we get

(30) 2≤||h||0≤||x∗||0+||x−||0≤2k,1≤||h+||0≤k,1≤||h−||0≤k,

and the contradiction. We conclude the proof.

Now we are ready to give the main result of this paper, which is called the nonnegative restricted property.

**Theorem 11**

Assume that *k* ≥ 1 is such that

(31) $$\begin{array}{c} \delta_{2k}^{+}+\left(\sqrt{2}+1\right)\theta_{k,2k}^{+}<1, \end{array}$$

and x* ∈ *𝒮* with ||x*||_0_ = *k*. Then x* is exactly the common unique minimizer of ((*P* _0_)) and ((*P* _1_)).

**Proof**

Since (31) implies *δ*_*k*_^+^ + *θ*_*k*,*k*_^+^ < 1, sol(*P*_0_) = {x*} by Theorem 10.

Suppose that $\overset{-}{x}$ is a solution of ((*P* _1_)). Take $h=\overset{-}{x}-x^{*}$. To get sol(*P*_1_) = {x*}, it suffices to verify that *h* = 0. The proof includes three steps, the first two steps are parallel to that in [19], in the third step, we utilize the technique of projecting the null space of *A* on ℝ_+_^*n*^; for details, see (42) and the argument around it.

Firstly, we introduce a partition of {1,…, *n*}. Let *T*_0_ be the support set of x*, *T*_1_ the index set including the first *k* large components of $\overset{-}{x}$ in *T*_0_^*c*^, *T*_2_ the index set including the next *k* large components of $\overset{-}{x}$ in *T*_0_^*c*^∖*T*_1_, and so on. Thus,

(32) |Tj|=k, j=0,1,…,[nk]−1,|Tj|≤k, j=[nk].

Moreover, for any *j* = 0,1,…, [*n*/*k*], we define

(33) $$\begin{array}{c} {\left(h_{T_{j}}\right)}_{i}=\{\begin{array}{cc} h_{i}, & i\in T_{j},\\ 0, & \text{else}, \end{array} \end{array}$$

which is exactly that for any *j* = 1,…, [*n*/*k*]

(34) $$\begin{array}{c} {\left(h_{T_{j}}\right)}_{i}=\{\begin{array}{cc} {\overset{-}{x}}_{i}-x_{i}^{*}, & i\in T_{0},\\ {\overset{-}{x}}_{i}, & i\in T_{j},\\ 0, & \text{else}. \end{array} \end{array}$$

Therefore, *Ah* = 0, *h* = ∑_*j*=0_^[*n*/*k*]^ *h*_*T*_*j*__, and

(35) hTj≥0, j=1,…,[nk],||hTj||0≤k, j=0,1,…,[nk].

Next, we show that ||*h*_(*T*_0_∪*T*_1_)^*c*^_||_2_ is bounded by ||*h*_*T*_0__||_1_. Note that for each *j* ≥ 2,

(36) $$\begin{array}{c} {||h_{T_{j}}||}_{2}\leq \sqrt{k}{||h_{T_{j}}||}_{\infty }\leq \frac{1}{\sqrt{k}}{||h_{T_{j-1}}||}_{1}, \end{array}$$

where the second inequality is because of the monotonicity of *h*_*i*_ on *T*_0_^*c*^, and

(37) $$\begin{array}{c} \sum_{j=2}^{\left[n/k\right]}{||h_{T_{j}}||}_{2}\leq \frac{1}{\sqrt{k}}\sum_{j=1}^{[n/k]-1}{||h_{T_{j}}||}_{1}\leq \frac{1}{\sqrt{k}}{||h_{T_{0}^{c}}||}_{1}. \end{array}$$

This gives

(38) $$\begin{array}{c} {||h_{{\left(T_{0}\cup T_{1}\right)}^{c}}||}_{2}={||{\sum_{j=2}^{\left[n/k\right]}h}_{T_{j}}||}_{2}\leq \sum_{j=2}^{\left[n/k\right]}{||h_{T_{j}}||}_{2}\leq \frac{1}{\sqrt{k}}{||h_{T_{0}^{c}}||}_{1}. \end{array}$$

In fact,

(39) ||xT0∗||1=||x∗||1≥||x−||1=||x∗+h||1=||xT0∗+hT0||1+||xT0c∗+hT0c||1≥||xT0∗||1−||hT0||1+||hT0c||1−||xT0c∗||1=||xT0∗||1−||hT0||1+||hT0c||1,

which implies

(40) $$\begin{array}{c} {||h_{T_{0}^{c}}||}_{1}\leq {||h_{T_{0}}||}_{1}. \end{array}$$

By applying (38) and (40), we have

(41) $$\begin{array}{c} {||h_{{\left(T_{0}\cup T_{1}\right)}^{c}}||}_{2}\leq \frac{1}{\sqrt{k}}{||h_{T_{0}^{c}}||}_{1}\leq \frac{1}{\sqrt{k}}{||h_{T_{0}}||}_{1}. \end{array}$$

Finally, we show that ||*h*_*T*_0_∪*T*_1__||_2_ = 0. By utilizing the projection *h* = *h* ^+^ − *h* ^−^, where *h* ^+^ and *h* ^−^ are projections of *h* and −*h* on ℝ_+_^*n*^, we have *h*_(*T*_0_∪*T*_1_)_ = *h*_(*T*_0_∪*T*_1_)_^+^ − *h*_(*T*_0_∪*T*_1_)_^−^. Moreover,

(42) $$\begin{array}{c} {\left(h_{\left(T_{0}\cup T_{1}\right)}^{+}\right)}_{i}=\{\begin{array}{cc} {\left({\overset{-}{x}}_{i}-x_{i}^{*}\right)}_{+}, & i\in T_{0},\\ {\overset{-}{x}}_{i}, & i\in T_{1},\\ 0, & \text{else}, \end{array}\\ {\left(h_{\left(T_{0}\cup T_{1}\right)}^{-}\right)}_{i}=\{\begin{array}{cc} {\left({\overset{-}{x}}_{i}-x_{i}^{*}\right)}_{-}, & i\in T_{0},\\ 0, & i\in T_{1},\\ 0, & \text{else}. \end{array} \end{array}$$

It is easy to see

(43) ||h(T0∪T1)+||0≤2k,||h(T0∪T1)−||0≤k.

From *Ah* = 0, we compute

(44) ||Ah(T0∪T1)||22=〈Ah(T0∪T1),Ah−Ah(T0∪T1)c〉=−∑j=2[n/k]〈Ah(T0∪T1),AhTj〉.

On one hand, based on the definition of NROC, (35), and (43), for *j* ≥ 2,

(45) −〈Ah(T0∪T1),AhTj〉 =−〈Ah(T0∪T1)+,AhTj〉+〈Ah(T0∪T1)−,AhTj〉 ≤|〈Ah(T0∪T1)+,AhTj〉|+|〈Ah(T0∪T1)−,AhTj〉| ≤θk,2k+||h(T0∪T1)+||2·||hTj||2  +θk,k+||h(T0∪T1)−||2·||hTj||2 ≤θk,2k+||hTj||2·(||h(T0∪T1)+||2+||h(T0∪T1)−||2) ≤2θk,2k+||hTj||2·||h(T0∪T1)||2,

where the last inequality is by the fact that (*a* + *b*)^2^ ≤ 2(*a* ^2^ + *b* ^2^). On the other hand, together with the definition of NRIC and (43), one has

(46) ||Ah(T0∪T1)||22 =||Ah(T0∪T1)+−Ah(T0∪T1)−||22 =||Ah(T0∪T1)+||22+||Ah(T0∪T1)−||22  −2〈Ah(T0∪T1)+,Ah(T0∪T1)−〉 ≥(1−δ2k+)||h(T0∪T1)+||22+(1−δk+)||h(T0∪T1)−||22  −2θk,2k+||h(T0∪T1)+||2·||h(T0∪T1)−||2 ≥(1−δ2k+)||h(T0∪T1)+||22+(1−δk+)||h(T0∪T1)−||22  −θk,2k+(||h(T0∪T1)+||22+||h(T0∪T1)−||22) =(1−δ2k+−θk,2k+)||h(T0∪T1)+||22  +(1−δk+−θk,2k+)||h(T0∪T1)−||22 ≥(1−δ2k+−θk,2k+)||h(T0∪T1)||22.

Therefore, we compute

(47) (1−δ2k+−θk,2k+)||h(T0∪T1)||22 ≤2θk,2k+||h(T0∪T1)||2  ·∑j=2[n/k]||hTj||2 (by  (44),  (45),  and  (46)) ≤2θk,2k+||h(T0∪T1)||2·1k||hT0c||1 (by (37)) ≤2θk,2k+||h(T0∪T1)||2·1k||hT0||1  (by (40)) ≤2θk,2k+||h(T0∪T1)||2·||hT0||2 ≤2θk,2k+||h(T0∪T1)||22,

where the forth inequality is from the fact that ||*h*_*T*_0__||_1_^2^ ≤ *k*||*h*_*T*_0__||_2_^2^. Then the assumption $\delta_{2k}^{+}+(\sqrt{2}+1)\theta_{k,2k}^{+}<1$ forces

(48) $$\begin{array}{c} {||h_{\left(T_{0}\cup T_{1}\right)}||}_{2}^{2}=0. \end{array}$$

Thus,

(49) $$\begin{array}{c} h_{\left(T_{0}\cup T_{1}\right)}=0. \end{array}$$

Therefore, we get *h*_(*T*_0_∪*T*_1_)^*c*^_ = 0 by (41), hence ||*h*|| = 0. This is exactly what we want. We complete the proof.

## 5. Conclusion

In this paper, we have derived a nonnegative restricted property condition, which ensures the exact recovery of sparse nonnegative image/signal via the linear program relaxation. Since the NRIC and NROC are defined in ℝ_+_^*n*^, there may be more types of measurement matrices satisfying the nonnegative restricted property than that in the case of RIP, regardless of random matrices or deterministic matrices. As a byproduct of the main result, we have investigated the solution property of the sparse nonnegative recovery and shown that any solution of ((*P* _0_)) must be one of the extreme points of its feasible set. However, it is not clear whether a given extreme point of the feasible set is a solution to ((*P* _0_)). This can serve as a target for future work.
